# How we perceive our own retina

**DOI:** 10.1098/rspb.2017.1904

**Published:** 2017-10-25

**Authors:** Kuno Kirschfeld

**Affiliations:** Max-Planck-Institute for Biological Cybernetics, Spemannstrasse 41, 72076 Tuebingen, Germany

**Keywords:** retina, perception, neural activity

## Abstract

Ever since the days of René Descartes, in the seventeenth century, the search for the relationship between subjective perception and neural activity has been an ongoing challenge. In neuroscience, an approach to the problem via the visual system has produced a paradigm using perceptual suppression, changing with time. Cortical areas in which the neural activity was modulated in temporal correlation with this percept could be traced. Although these areas may lead directly to perception, such temporal correlation of neural activity does not suffice as ultimate proof that they actually do so. In this article, I will use a different method to show that, for the perception of our own retina, any brain area leading directly to this perception also needs to represent the retina without distortion. Furthermore, I will demonstrate that the phenomenon of size constancy must be realized in this area.

## 1. Background

**Figure 1.**
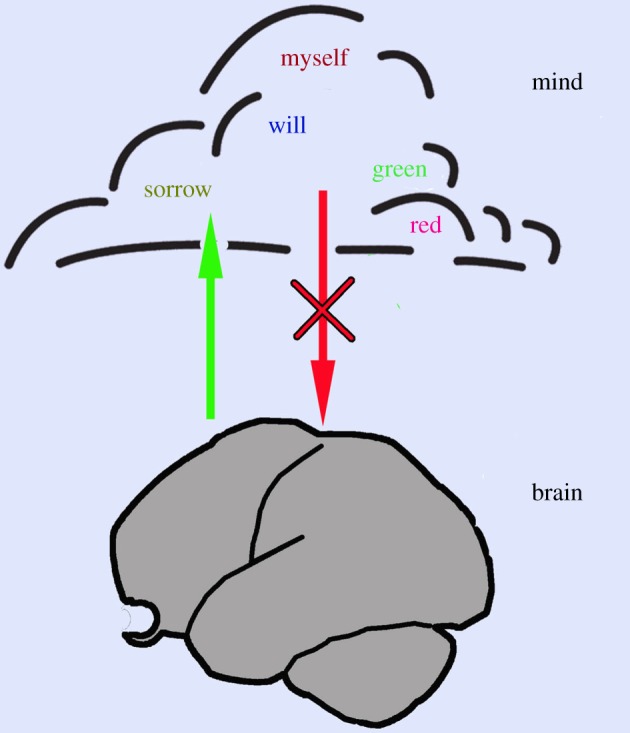
Relationship between brain and mind. The mind is qualitatively different from the brain in that what we become aware of in our mind cannot be verified objectively with science-based methods. The content of the mind is accessible to subjects only, and is therefore also called the ‘inner perspective’. Although the brain generates everything that goes on in the mind (green arrow), no influence from the mind on the brain (i.e. from outside the world of physics) has ever been demonstrated (crossed red arrow). (Online version in colour.)

Two decades ago, Francis Crick in his book *The astonishing hypothesis* [1] came to the conclusion that the brain generates everything that goes on within our mind: ‘that “You”, your joys and your sorrows, your memories and your ambitions, your sense of personal identity and free will, are in fact no more than the behavior of a vast assembly of nerve cells and their associated molecules' (p. 3). He proposes that, besides the brain, we also have something qualitatively different: a ‘soul’ or a mind. In figure 1, the brain and the mind are depicted as lying one above the other. As we cannot localize the mind, it is depicted as a cloud. It is a question of how both interact.

According to Crick, everything that takes place within our mind originates in the brain. In other words, the brain affects the mind. This statement is endorsed by, among others, the following observations. Whenever modifications of the brain occur, the mind may be modified as a result. If a part of the brain is destroyed by a stroke, deficits may ensue, for instance in perception. The green arrow in figure 1 symbolizes this relationship and raises the question as to which part of the brain creates consciousness or, as Crick [1] puts it: ‘Where are the “awareness neurons”—are they in a few places or all over the brain—and do they behave in any special way?’ (p. 204).

This is the question of the neural correlate of awareness, which was later termed the ‘neural correlate of consciousness’ (NCC). This question was the subject of a number of papers by Francis Crick and Christof Koch [2,3]. They came to the conclusion that the best way to approach the problem of consciousness is to study the visual system in both man and his close relations due to our unique knowledge of the structure and function of their visual systems.

**Figure 2.**
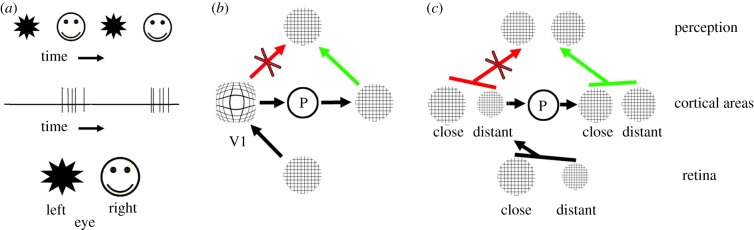
Schematic relationships between retina, cortical areas and perception under different experimental situations. (*a*) The left eye looks towards a star, the right one towards a face. In this situation, it is not a superposition of both images that is perceived, but the star and the face in alternation. Monkeys can be trained to pull a lever to indicate when they perceive, for example, the face. In certain brain areas, neurons could be shown to generate spike activity in temporal correlation with face perception. (*b*) A regular grid on the retina is distorted in the primary visual cortex, area V1. As the perception is undistorted before perception, processing (P) must take place to compensate for the distortion. The arrows indicate the viable (green) and non-viable transitions between brain and perception. (*c*) The retinal image of a close object is large (left), while that of a distant one is smaller (right). In any retinotopic area without size constancy, the close and distant objects will be represented as different in size. In our perception, we experience size constancy. Before objects represented in areas without size constancy can become consciously perceived, processing (P) must take place to generate size constancy. The arrows indicate the possible transitions between brain and perception. (Online version in colour.)

In certain experimental paradigms, the perception of an object can be suppressed even if its image on the retina remains constant. This can be shown in experiments of *binocular rivalry*, in which, for example, a face is projected to one eye while a star is projected to the other [4]. Rather than perceiving one object as being superimposed on the other, the percept of the two images alternates in time. One way of specifying the NCC is to determine a temporal correlation between neural activity and the percept of an object. It transpires that neural activity in certain cortical areas can be modulated synchronically with perceptual reports (figure 2*a*). While such a temporal correlation can be considered a necessary prerequisite for the NCC, it is not sufficient to prove that a particular cortical area acts as an NCC. Many laboratories have investigated what is believed to be the neural correlate of consciousness (reviews in [4–9]), which is also the topic of this paper. Further approaches have been used to specify properties of NCCs, such as motion-induced blindness, visual masking or flash suppression [9].

## 2. Method

**Figure 3.**
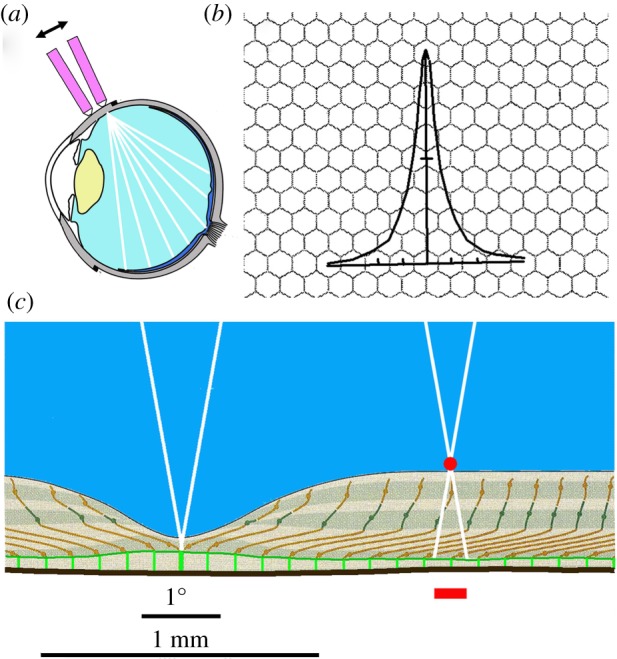
(*a*) The method of retrolental illumination. A small LED lamp (lens diameter smaller than 5 mm) is used to illuminate the eye from behind the lens. It needs to be moved (double arrow) to overcome fading of the observed image due to stabilized image conditions. (*b*) Illustration of the blurring effect of the human lens. The line spread function of the human eye (pupil diameter 2.4 mm [23]) is superimposed on the (idealized) foveal receptor raster. The labelling on the abscissa is 1 min of arc. Neighbours of the receptor in the centre of the line image still receive some 20% of the light intensity of the latter. (*c*) Cross section through the human retina with light rays coming from different directions (redrawn from [10]). The black line at the bottom is the pigment epithelium. The thick green rods orthogonal to the pigment epithelium are the inner and outer segments of the photoreceptors which act together as light guides (for clarity, only a small percentage was drawn). The vitreous body is shown in blue. Different retinal layers between the pigment epithelium and the vitreous body are indicated. The red point in the right half of the figure illustrates the cross section of a blood vessel located at the border between the retina and the vitreous body. If light rays enter from different directions (white lines), the image of the blood vessels falls on different photoreceptors, whereby it is shifted relative to the foveal receptors, which stay stable.
  The lamp has to be moved steadily a few millimetres in different directions to overcome adaptation which, in normal vision, conceals the retinal blood vessels: The vessels are stabilized on the retina, and stabilized images disappear in seconds. Saccades are irrelevant in this case because the retinal image moves in accordance with any eye movement.
  To estimate, for example, the diameter of the fovea, we placed a piece of grey cardboard at a distance of about 30 cm from the eye of the observer. Dockets corresponding to diameters of 1.5, 2.0 and 2.5 degrees were attached to the cardboard. One eye was illuminated by RLI; by opening the other eye and observing the dockets on the piece of cardboard, the diameter of the fovea, among other things, could be estimated. A wide range of illuminances of the cardboard, from 0.5 to 500 cd m^−2^ can be used.
(2) We will compare the image of the retina, as taken, for example, by a fundus camera, with our own conscious perception of the retinal image. Differences between these two images would reveal what kind of processing takes place before we become aware of the retina's image. In this context, it is particularly interesting to bear in mind what we would expect if area V1 directly contributed to visual awareness. This is because area V1 constitutes a bottleneck of visual signals on their way from the retina to the cortex. Whether or not it makes a direct contribution to our awareness has long been the subject of discussion [4–9].
(3) I will make use of the mind's inability to process information. This is due to the fact that information processing requires a physical, material substrate that the mind does not have at its disposal. Before information can be processed, it must first be stored and, even at this stage, a material substrate is essential.

My considerations are based on the following three issues:

(1) Our eyes are constantly in motion and hence the image on the retina is virtually never stable. This is particularly obvious when we look at an extended object, such as a face, which is scanned by a number of saccades that rapidly alter the direction of our gaze. At each saccade, we take an individual ‘snapshot’, as it were, of the optical target. These different snapshots are processed within the visual system to produce what we then perceive as a face [10]. It thus seems worth making the effort to identify a single ‘snapshot’ and its conscious perception before its information is processed any further.
  If we consciously perceive our own retina, it is like looking at such a snapshot. It is possible to observe one's own retina by illuminating the eye side-on. In this case, light enters the eye behind the lens (‘retrolental illumination’, RLI; figure 3*a*) and the retinal vessels throw a shadow onto the photoreceptors so that the subject perceives a wonderful view of his own vessels, known as the entoptic image of the retina. This phenomenon was first described by Purkinje [12]. It is elicited by focusing light from the sun or from a penlight onto the sclera. In addition to the laser output, many modern laser pointers have an LED light whereby the phenomenon can be easily generated. We used a Zehui 3-in-1 Laserpointer from Amazon (Germany). The intensity of the LED lamp is not indicated by the manufacturer.

## 3. Results

### (a) The perceived image is undistorted

**Figure 4.**
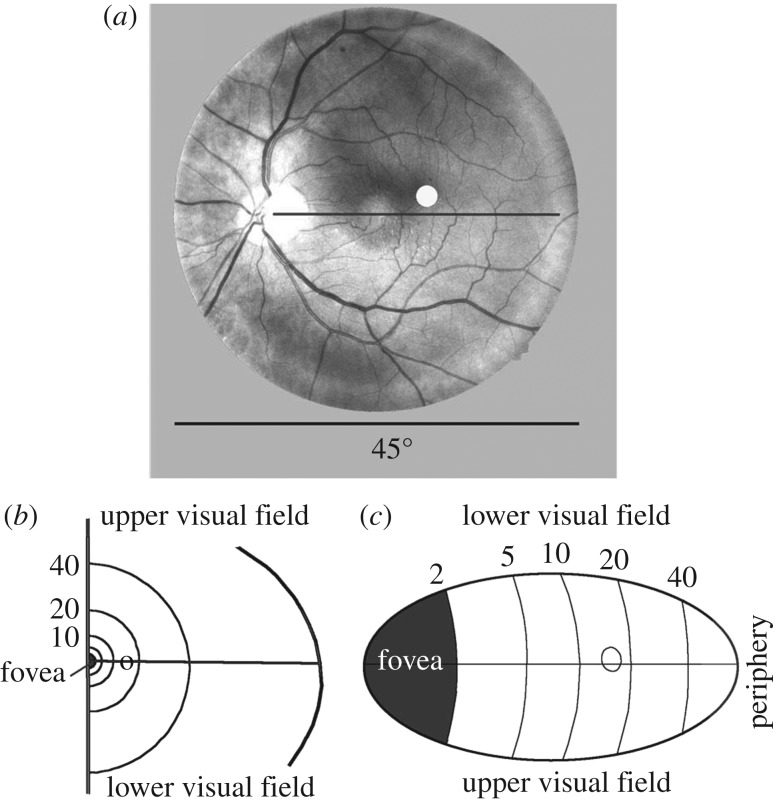
Retina and area V1. (*a*) Fundus photograph of my left eye. Photograph taken by a Kowa Nonmyd 7 non-mydriatic fundus camera. The plotted white point indicates the location of the fovea. (*b*) Half of the visual field of the right eye. (*c*) Left striate cortex. Numbers indicate degrees; the circles indicate the location of the blind spot. (*b*) and (*c*) according to Rodieck [10], modified.

As in the fundus photograph shown in figure 4, the arteries and veins and their convergence to the optic disc can be observed by retrolental illumination. If we compare fundus photographs and drawings from observers from their entoptic images of the retina, we gain the impression that the entoptic images are not distorted [13]. However, these entoptic images were scaled, rotated and translated to achieve maximum correspondence in the central fovea. We therefore looked for independent information on the question of whether or not the entoptically perceived image is distorted. In this context, the fovea is of particular interest because its size in the retina and its representation in area V1 differ significantly: in the retina, the fovea is small compared, for example, with the size of the blind spot, whereas in area V1 it is exaggerated (figure 4*b*,*c*).

When RLI is used, the fovea is seen as a grainy spot in the area without vessels. On account of the grains, v. Helmholz [14] described this area as having the appearance of ‘shagreen leather’. Although its diameter can be estimated, it cannot be measured with high accuracy for two reasons: (i) the border of the anatomical fovea is not precisely defined (figure 3*c*) and (ii) the permanent motion of the LED lamp introduces a certain degree of instability. High accuracy is, however, not necessary, because the main aim of the investigation is to discriminate between the two options: Does what we perceive correspond to the retina, or is the fovea accentuated as in area V1? The difference between these two alternatives is so striking that high precision is not required to discriminate between them (figure 4*b,c*).

With the method described above, observers had to decide whether the size of the grainy spot corresponds best to a docket of 1.5, 2.0 or 2.5 degrees; all four observers found the best coincidence with the 2 degrees spot. This meets our expectations according to histology (figure 3*c*).

If the LED lamp is moved, the grainy area can be seen to move in relation to the vessels. This is a necessary consequence of the fact that vessels and receptors are not located on the same plane. As illustrated in figure 3*c*, when the direction of incidence of the light is altered, the image of the vessels is displaced in relation to that of the photoreceptors. Given the anatomical dimensions of the retina, a displacement of the light incidence of ±10° leads to a relative displacement between the fovea and the vessels of approximately a quarter of a degree (figure 3*c*). This is qualitatively in agreement with the observation in our experiments.

A second obvious parameter of the perceived retina is the diameter of the fovea compared with the blind spot. Even if it is not possible to draw the entoptically perceived pattern of vasculature with high accuracy [13], all four observers reported that what they perceive looks very similar to figure 4*a*. In particular, it is obvious that the fovea is smaller than the blind spot. This is different from what would be expected if perception were to correspond to the representation in V1: In this case, the fovea should be significantly larger than the blind spot (figure 4*c*).

This means that the perception of our retina is not severely distorted in comparison with its structure as represented on the fundus photograph. Nor does this seem to correspond to what we would expect if area V1 were to contribute directly to the entoptic perception of the retina.

As the photograph taken by the fundus camera is the projection of the spherical retina onto the flat camera sensor, a certain amount of distortion is to be expected. However, in our vision, the distortion of plane images becomes obvious only for large angular extensions [14]. The fundus photograph of the retina (figure 3*a*) covers a mere 45°, an area in which there is only a small deviation from linearity.

### (b) The retina differs from its percept

To estimate the angular extent of the fovea, we observed our retina superimposed on a piece of grey cardboard. During this experiment, it became clear that the size of the perceived retina is not constant, but that it varies according to the distance between the piece of cardboard and the eye: the greater the distance, the larger is the image. This is at variance with the fundus photograph, the size of which appears more or less constant, even if we change the distance from which we look at it. It has already been well established that the apparent size of the vascular tree depends on the distance judgement of the background onto which it is projected [15].

**Figure 5.**
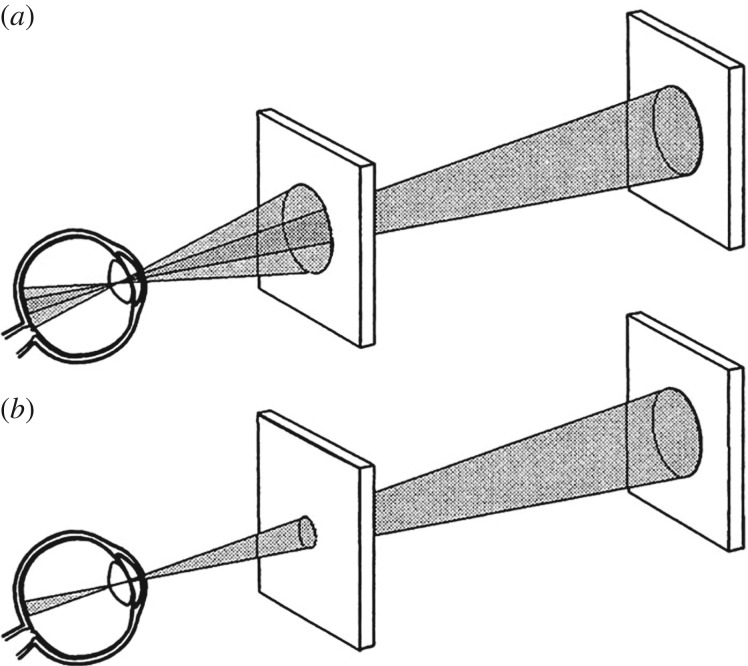
Illustration of Emmert's law. (*a*) Natural viewing condition. The further away a real object of constant size is situated, the smaller it is imaged on the retina. Nevertheless, to us it appears constant in size irrespective of its distance. This is due to the fact that, before we become aware of the object, the brain takes its distance into account by amplifying the object's representation in a primary visual area by a factor that increases as the distance increases. (*b*) An after-image on the retina remains constant in size irrespective of whether it is observed on a close or a distant screen. The greater the distance of the screen from the eye, the larger the after-image appears. This is because, here too, the brain takes the distance of the screen into account by amplifying the object's representation in a primary visual area by a factor that increases together with the distance. This explanation was provided by Emmert [16]; the relationship is known as *Emmert's law.*

This phenomenon is explained in figure 5 in the context of after-images. Figure 5*a* shows what happens when we look at a real object. The size of the retinal image of such an object depends on its distance from the eye. Irrespective of the different sizes of the retinal images, the size of the object appears fairly constant to us. This is due to the mechanism of size constancy which, under natural viewing conditions, comes into play before we become aware of the object. This mechanism takes the distance of the object into account and corrects the size of the perceived object accordingly. If an object is relatively close (i.e. within our arm span of up to half a metre), the mechanisms of lens accommodation and eye convergence contribute the required information to approximately the same extent [17]. For greater distances, further parameters, such as precognition of the size of known objects or the turbidity of the air, come into play.

As illustrated in figure 5*b*, if the same mechanism has an impact before we perceive an after-image, then the consciously perceived image will be all the larger, the greater the distance of the plane on which it is observed. This phenomenon was detected by Emmert [16]. Similar to after-images, the vascular tree on the retina is also of constant size, and hence Emmert's law holds here also [15]. Whenever the possibility of estimating the distance of an object is impaired (by, for example, the application of a mydriaticum which blocks accommodation), size constancy and Emmert's law are modified accordingly, but perception is not prevented.

### (c) Necessary properties of an neural correlate of consciousness

We can now specify the conditions required for a cortical area to be considered to lead directly to perception (i.e. to be an NCC). Let us first consider the primary visual cortex, area V1. We know that any visual object in V1 is represented retinotopically (i.e. neighbourhood relationships are maintained). However, we also know that this representation is significantly distorted: 1% of the retina (corresponding to a diameter of 7° around the fovea) occupies about 50% of V1's area [10,18]. The distortion has been quantified by a magnification factor, which is a function of eccentricity [19]. To demonstrate this distortion, which is illustrated schematically in figure 2*b*, I have used a regular grid as the image on the retina. In area V1, the grid is retinotopically represented, albeit in a distorted fashion: the area around the central fovea is exaggerated. Area V1 is considered a bottleneck that has to be passed by all visual information leading to perception [4–9]. This implies that, before perception can take place, the V1 representation has to be processed so as to compensate for the distortion. Because the mind cannot process information, this processing must occur within the brain. The situation is illustrated schematically in figure 2*b*: This ‘processing’ occurs in the cortical element P. This is meant in a rather general way and not only refers to computation, but can also stand for anatomical divergence or convergence of axons. However, because all processing takes place within the brain, and not in the mind, V1 per se cannot be the NCC. As indicated on the right in figure 2*b*, there must therefore be an area that represents the grid in an undistorted fashion.

When discussing the role of any area as a possible NCC, a second fact must also be taken into consideration, namely that, in perception, we encounter the phenomenon of size constancy. The size of a retinal image of a real object depends on its distance from the eye, whereas perception, under normal conditions, is constant in size. This situation is depicted in figure 2*c*. I also used this argument when discussing the NCC in the context of after-images [20].

There is no question that, in area V1, the retina is represented in a distorted way (figure 4). Unexpectedly, recently it has been shown that the retinotopic activity in V1 associated with viewing an after-image is modulated by perceived size, even if the size of the retinal image remains constant. This suggests that V1 has an important role in size constancy [21].

## 4. Discussion

The conclusion that the perception of our entoptically perceived retina is undistorted does not come unexpectedly: we have to act within the real world, which would be difficult without an undistorted representation of this world. It would also lead to a contradiction of the image of the world that we gain from our sense of touch. But what, then, is the function of area V1 with its distorted representation? The situation is reminiscent of the somatosensory ‘homunculus’. Here too, the representation is distorted: the higher the spatial touch resolution in an area of the skin, the larger the cortical area it occupies. Our perception is not distorted here either. We perceive our lips as they are, and not exaggerated as in the somatosensory cortex. It may be that, within area V1 and within the somatosensory cortex, the information emanating from the sense organs is stored (and to some degree preprocessed) in such a way that each resolved pixel occupies a cortical surface element of similar size. From here, this information can then be called up for different functions.

One conspicuous phenomenon of the perceived foveal area under retrolental illumination is that it appears ‘grainy’. When he described the foveal area as having the appearance of ‘shagreen leather’, v. Helmholz [14] already considered the possibility that this was due to the activity of individual cones. Ehrich [22] later estimated that the number of grains corresponds to approximately 1000, which is less than the number of cones in this area. However, when the light source is moved, some grains disappear, while others appear, and hence he concludes that the grains do indeed correspond to the activity of single cones.

The question then is: why can we see individual pixels in retrolental illumination but not in real vision? One plausible answer is that, in real vision, light has to pass the lens of the eye, which causes a certain amount of blurring. This means that it is not possible to illuminate an individual photoreceptor without also illuminating its neighbours. The contrast between the signals of neighbouring photoreceptors under normal viewing conditions will therefore be reduced. Figure 3*b* illustrates the distribution of cones in the fovea, separated by 1 min of arc [11]. The distribution of illuminance occurring on the retina from a narrow line source of light (line spread function) at a pupil diameter of 2.4 mm, being the best possible case (smaller and wider pupils lead to broadened distributions) [23], is superimposed on the figure. Even in this optimal case, however, blurring is considerable and contrast reduction for fine structures may therefore be too strong for the detection of individual receptors. By applying retrolental illumination, the lens can be bypassed and its blurring eliminated. The directional sensitivity of individual photoreceptors [24] causes the movement of the LED lamp to modify the amount of light that is absorbed in each photoreceptor and, as such, to overcome the stabilized image fading.

The hypothesis that foveal ganglion cells have receptive field centres that are fed by single cones is supported by psychophysical experiments using laser interference fringes which—as in retrolental illumination—remain unaffected by diffraction and aberrations of the eye's optics [25].

How does the conclusion drawn in this paper on the properties of NCC mediating the perception of our own retina under RLI fit into more general concepts on consciousness? Dennett & Kinsbourne discussed two alternative models of consciousness: the ‘Cartesian theater’ model and the ‘multiple drafts’ model [26]. According to the first, there is a place in the brain where ‘it all comes together’ and is ‘presented’ for subjective statement. According to the second, ‘discriminations are distributed in both space and time in the brain' [26, p. 183]. In both models, perception of complicated processes are considered; for example, what we perceive if, after a red flash, a green flash appears a few degrees apart (color phi motion). The topic of the present paper considers a simpler case, the perception of our own retina under RLI, without any temporal aspects. The result presented here cannot contribute to the question of whether a Cartesian theatre model or a multiple drafts model is realized: It would be compatible with either of them. The same is true for other general models of conscious perception of visual stimuli, such as that of Lamme [27] (widespread recurrent processing between visual cortical areas as well as with the frontoparietal network) or that of Dehaene and co-workers [28] (entry of processed visual stimuli into a global brain state linking distant brain areas). They are neither supported nor rejected by my result.

The assertion that the mind is unable to process information, which is crucial for the conclusions drawn above, is implicitly included in Crick's statement quoted in the first paragraph. It also contains the premise that the mind cannot influence the brain. All processes in the brain are subject to the laws of physics and chemistry, and no influence on the brain from the mind (i.e. from outside this world) has ever been verified. For this reason, the red arrow in figure 1 is crossed out. This also implies that the mind cannot ‘think’, because thinking is the processing of information. However, the concept that the mind is unable to think and that only the brain is capable of doing so goes against our intuition. In this respect, Crick's hypothesis is indeed astonishing. The other consequence is that our ‘will’ is not able to initiate an action but that actions are instead initiated by our brain, which later also generates the perception of our volition. This has been shown in experiments by Benjamin Libet and co-workers, and is widely discussed in the context of the problem of ‘free will’ [29].

## Observers

Three observers as well as the author verified the described observations by retrolental illumination. All observers gave their written informed consent to the experiments. One observer could specify the fovea, but was unable to observe the ‘grains’.

## Data accessibility

Statistical evaluation of data was not necessary.
